# Prevalence of human papillomavirus in penile malignant tumors: viral genotyping and clinical aspects

**DOI:** 10.1186/s12894-015-0007-8

**Published:** 2015-02-24

**Authors:** Isaura Danielli Borges de Sousa, Flávia Castello Branco Vidal, João Paulo Castello Branco Vidal, George Castro Figueira de Mello, Maria do Desterro Soares Brandão Nascimento, Luciane Maria Oliveira Brito

**Affiliations:** Tumors and DNA Bank of Maranhão, Federal University of Maranhão (UFMA), São Luís, Brazil; Department of Morphology, Federal University of Maranhão (UFMA), São Luís, Brazil; José Alencar Gomes da Silva National Cancer Institute, Department of Genetics, Rio de Janeiro, Brazil; Maranhão State Institute of Oncology Aldenora Bello (IMOAB), São Luis, MA Brazil; Department of Medicine III, Federal University of Maranhão (UFMA), São Luís, Brazil

**Keywords:** Papillomavirus Infections, Penile Neoplasms, Association, Men’s health

## Abstract

**Background:**

The human papillomavirus (HPV) prevalence in males has been reported to be between 3.6% and 84%, depending specially on the socioeconomic status. HPV infection has been related as a risk factor for penile cancer. This is a rare tumor, and other risk factors include lack of personal hygiene and men who have not undergone circumcision. Penile cancer is less than 1% of cancers in men in the United States, however, is much more common in some parts of Asia, Africa, and South America, where it accounts for up to 10% of cancers in men. This study aimed to determine the prevalence of HPV-DNA in penile cancers in São Luís, Brazil and to correlate the virus presence to histopathological factors.

**Methods:**

Tumor paraffin samples of 76 patients with penile carcinoma were tested in order to establish the prevalence and distribution of genotypic HPV using PCR/Nested and automated sequencing. To evaluate the association between HPV types and other clinical and morphological variables, a nonparametric ANOVA was performed using a Kruskal Wallis test, and statistical significance was determined to a value of p < 0.05.

**Results:**

The average age of patients at the time of diagnosis was 66 years ± 17.10. Regarding location, 65.79% of the tumors were located in the glans, and the most common types were vegetative (34.21%) and squamous (98.68%). Most of the lesions ranged in size from 2.1 to 5.0 cm, presenting Jackson I stage and Broders II degree. It was observed that 32 patients had at least one invaded and/or infiltrated structure. Lymph node involvement was observed in 19.76% of the patients, and 21.05% showed an inflammatory process. In the molecular evaluation, HPV infection was observed in 63.15% of the lesions, and the most common type was HPV 16.

**Conclusions:**

From the statistical analysis, it can be verified that the variables were not associated with infection by the HPV virus. Although penile cancer can result from various risk factors that act in synergy, an HPV virus infection is important for the development of such neoplasm.

## Background

The human papillomavirus (HPV) is a DNA-virus from the Papoviridae family - genre Papillomavirus, with more than 100 types currently recognized, 20 of which can infect the genital tract; the man is the main disseminator [1,2].

Penile infection by HPV may be clinical, subclinical or latent. In clinical presentation, the diagnosis is simpler, because it is determined from a good clinical examination to uncover existing lesions. In subclinical and latent forms, other methods, such as peniscopy, are necessary to aid in detection, as it is not possible to detect changes (i.e., diagnosis) with the naked eye. In men, there is a higher frequency of the subclinical form [2].

Penile cancer mainly affects men over 50 years old, but approximately 19% of patients are 40 years of age or younger, and 7% are below the age of 30 [3]. The major risk factors of the disease are associated with hygiene, phimosis, smegma retention, inflammation process, and HPV infection [4].

The prevalence of the virus in males has been reported to be between 3.6% and 84%, depending on socioeconomic status [5,6]. Penile cancer represents 0.4% to 0.6% of all malignant tumors in developed countries, such as the United States and European countries, and more than 10% of all malignant tumors in developing countries, such as those in Asia, Africa and South America [3,4].

According to Nardi et al. [7] the highest incidence rates of penile carcinoma were found in Maranhão. Maranhão is a city situated in the Northeast of Brazil. Favorito et al. [8] observed a predominance of reports of penile cancer in the North and Northeast (53.02%), which are regions with lower human development indexes. The understanding of HPV prevalence and knowledge of the viral subtype distribution constitute important epidemiological information that can assist the development of local or regional public policies to prevent HPV and of new vaccines.

The aims of this study were to detect and perform HPV genotyping in biological specimens of penile tumors and to determine the existing associations between viral presence and histopathological clinical aspects.

## Methods

### Enrollment

This was a retrospective study performed in paraffined penile tumors collected at two public reference hospitals in Maranhão. A total of 76 samples were included in the study from patients diagnosed with penile cancer between the years 2001 and 2011. Patient information as well as the histopathological characteristics of the tumors obtained from medical records. As the samples consisted in paraffined tumours, there was no written informed consent from the patients. The patient identity was not disclosed in this research. This work was approved by the Ethics in Research Committee of the University Hospital of the Federal University of Maranhão (HU/UFMA).

### Inclusion criteria

Paraffin blocks and histological slides of penile tumors as a result of biopsy or surgical treatment, with or without lymphadenectomy at any follow–up in the archives of the Pathology Services.

### Exclusion criteria

Histological slides and/or paraffined blocks not found in the archives of the Pathology Services of referral hospitals and reports did not provide complete information.

### HPV analysis

The samples were reviewed by the pathologist, and blocks with tumor representativeness (over 50% of the total area of the fragment) were selected. After microtomy, sections suffered a process of deparaffinization. The sections were stored at 4°C, awaiting DNA extraction.

The extraction of the genomic DNA from the samples was performed using the QIAamp DNA FFPE Tissue Purification Kit (QIAGEN®) according to the extraction protocol suggested by the manufacturer.

The Nested PCR reactions were performed by using primers PGMY09 and PGMY11 for the first round, and primers GP + 5 and GP + 6 for the second round [9].

The sequencing reactions were performed in the Laboratory of Genetics of the National Cancer Institute (INCA) with ET Dye Terminator Cycle Sequencing kit (GE Healthcare, UK) according to the manufacturer’s suggested protocol.

### Statistical analysis

All data were collected and prospectively input in an EpiInfo 3.4.3 and Microsoft Office 2007® were used for the statistical analysis.

To evaluate the association between HPV types and other clinical and morphological variables, a nonparametric ANOVA was performed using the Kruskal Wallis test with a statistical significance level of 5% probability (p < 0.05).

## Results and discussion

Tumor biopsies of penile cancer were evaluated in 76 patients aged 26 to 97 years with a mean of 60.7 years and standard deviation of ±17.10, presenting a higher prevalence in the over 66 age group. The clinical representation and pathologic characteristics distribution is shown on Table 1.

**Table 1:** **Age, clinical presentation and pathologic characteristics from 76 patients diagnosed with penile cancer**

**Age at diagnosis**	**N**	**%**
**Average age**	60.6 ± 17.10	-
26-45	16	21.05
46-55	12	15.79
56-65	16	21.05
66-97	32	42.11
**Lesion area**		
Glans add other regions	50	65.79
Foreskin	08	10.53
Corpus	03	3.95
Non evaluable	15	19.74
**Predominant morphology**		
Ulceration	17	22.37
Vegetating	26	34.21
Ulceration and Vegetating	17	22.37
Nodule and Vegetating	01	1.32
Non evaluable	16	21.05
**Size of the lesion (cm)**		
≤0,5	00	00
06-2,0	20	26.32
2,1-5,0	40	52.63
≥5,1	14	18.42
Non evaluable	02	2.63
**Staging of Jackson 1966**		
Stage I	33	43.42
Stage II	16	21.05
Stage III	11	14.48
Stage IV	16	21.05
**Broders’ Classification**		
Grade I	26	34.21
Grade II	36	47.37
Grade III	06	7.89
Non evaluable	08	10.53
**Invasion**		
Present	18	23.69
Absent	58	76.31
**Infiltration**		
Corpus add other regions	24	31.58
Perineural	01	1.32
Urethra	03	3.95
Stroma	03	3.95
Urethra and Stroma	01	1.32
Absent	44	57.89
**Lymph node involvement**		
Present	15	19.73
Absent	61	80.27
**Lymphatic embolization**		
Present	04	5.26
Absent	72	94.74
**Inflammatory process**		
Present	16	21.05
Absent	60	78.95

These results correspond with those obtained in the literature [10-14]. The average age of the patients at diagnosis predominates in advanced age (>50 years), which suggests that men seek health services very late in life [15]. Younger individuals are also affected, but in smaller percentages [7].

Regarding the location of the lesions, the glans, in an isolated form or associated with other regions, was the most affected structure as in the research by Delgado et al. [10], Wanick et al. [11] and Favorito et al. [8].

Studies have shown that the lesions on the glans are directly linked to poor hygiene. This occurs due to the formation of a mass, called smegma, followed by likely irritation of the site and onset of injury, facilitating various infections and future neoplasia if left untreated [10].

Regarding the clinical morphology, the predominantly found lesion was the vegetating type followed by ulceration. The occurrence of both types of lesions in the same patient was observed in 22.37% of the cases. In a study performed in Spain, researchers observed that the vegetative lesion was also more present, in 66% of the cases [12]. On the other hand, in another research conducted in Rio de Janeiro [10], a larger number of lesions was detected in the form of ulcerations, nearly 55.88% of the studied cases.

The dimensions of the lesions were similar to those observed in the Wanick et al. results [10], with a larger number of cases: 52.63% of the cases, with size between 2.1 and 5.0 cm.

Unlike other studies, the moderately differentiated tumors (grade II) identified in this work, according to Broder’s classification, were the most prevalent. Fonseca et al. [13] identified a greater number of cases classified as well differentiated (grade I). However, Scheiner et al. [14] observed higher incidence of grade III (undifferentiated) tumors, which can be explained by the greater presence of stage III and IV patients.

The findings indicated that invasion was present in 23.68%, and infiltration occurred in at least one of the structures, with the highest prevalence in the corpus cavernosum. Koifman et al. [15] reported the presence of invasion of the spongiosum or cavernous corpus in 41.3% of the patients.

Regarding lymph node involvement, a percentage of 19.73% was observed. According to Sacoto et al. [12], patients with more advanced disease and positive lymph nodes at the time of diagnosis had a worse survival rate than those with localized stages.

The DNA of the HPV was detected in 63.15% (48/76) of the samples. The oncogenic risk distribution is shown on Table 2. This percentage is within the range reported in the literature, which shows that the rate of HPV infection in penile malignant tumors may vary from 20 to over 75% of cases [16]. According to a systematic review of the prevalence of HPV in invasive tumors of the penis, 48% of the samples presented HPV infection [17]. A Belgian study by D’Hauwers et al. [18], which had the same number of patients as in this study, revealed that 70.9% of the tumors had the HPV virus. However, a survey conducted in Vietnam demonstrated that only 23% of tumors had HPV infection [19]. A study conducted in Brazil showed that 75% of invasive penile tumors were infected by HPV [14]. These variations may be due to different techniques used for viral detection, regional differences or histological type of the analyzed tumor.

**Table 2:** **HPV prevalence and distribution according to oncogenic risk in 76 patients diagnosed with penile cancer**

**HPV**	**n**	**%**
HPV +	48	63.15
HPV -	28	36.85
**Oncogenic risk**		
High risk	17	35.42
Low risk	6	12.50
Indeterminate	25	52.08

In our study, among the high-risk viral types present were the 16, 18, 45 and 69 types. The HPV of type 11 was the only low oncogenic risk found. Type 16 was the most prevalent, found in 10 cases, followed by type 11 of low risk with 6 cases, type 18 with 4 cases, type 69 with two cases and type 45 with 1 case. The automated sequencing technique was not effective for viral genotyping, because in more than 50% of the samples it was not possible to achieve. This may be due to the presence of co-infections in these samples, which prevents the device from detecting the virus, as described by Gharizadeh et al. (2005) and Verteramo et al. (2009) [20,21]. The most common viral type found in this study was HPV 16, high-risk type. This virus type was also the most found in other studies such as those developed by Do et al. [19] (89%), D’Hauwers et al. [18] (48.3%) and Heidman et al. [16] in (52%).

As shown in Table 3, no association was found (p < 0.05) between infection with HPV virus and clinical and histopathological and clinical variables, as was the case in the research by Do et al. [19], Fonseca et al. [22] and Scheiner et al. [14].

**Table 3:** **Association of clinical presentation and pathologic characteristics data with HPV infection patients with penile cancer**

	**HPV +**	**HPV -**	p-values*
**Lesion area**			0.543
Glans add other regions	31	19	
Foreskin	4	4	
Corpus	2	1	
Non evaluable	11	4	
**Predominant morphology**			0.377
Ulceration	11	06	
Vegetating	17	09	
Ulceration and Vegetating	09	07	
Nodule and Vegetating	01	00	
Non evaluable	10	06	
**Size of the lesion (cm)**			0.352
06-2,0	13	07	
2,1-5,0	23	17	
≥5,1	11	03	
Non evaluable	01	01	
**Histologic type**			0.285
Squamous	47	28	
Adenocarcinoma	01	00	
**Staging of Jackson 1966**			0.381
Stage I	21	12	
Stage II	07	09	
Stage III	07	04	
Stage IV	13	03	
**Broders’ Classification**			0.352
Grade I	20	06	
Grade II	21	15	
Grade III	02	04	
Non evaluable	05	03	
**Invasion**			0.578
Present	10	08	
Absent	38	20	
**Infiltration**			0.535
Corpus add other regions	15	9	
Perineural	0	1	
Urethra	3	0	
Stroma	3	0	
Urethra and Stroma	1	0	
Absent	26	18	
**Lymph node involvement**			0.285
Present	09	06	
Absent	39	22	
**Lymphatic embolization**			0.285
Present	04	00	
Absent	44	28	
**Inflammatory process**			0.285
Present	10	06	
Absent	38	22	

*Estimated by univariate logistic regression analysis;

P = Statistical significance; 95% CI = 95% confidence interval.

Infections by HPV are strongly associated with the development of penile cancer; however, the role of viruses in the etiology is not very clear [21]. Although the etiology is still unknown, approximately 40% of all penile tumors are related to HPV infection [22].

## Conclusion

HPV DNA was found in 48 of the 76 analyzed samples (63,15%). The high-risk type HPV 16 was observed in 21.28% (10/48) of the lesions followed by low-risk type HPV 11 in 12.76% (6/48) and high-risk types HPV 18 in 8.51% (4/48), HPV 69 in 4.25% (2/48) and HPV 45 in 2.13% (1/48). In 51.06% of the cases, genotyping was indeterminate, suggestive of co-infection.

The average age of the patients in the study was 60.6 years old. Prevalent lesions were larger than 2 cm, in the glans region, in general vegetating, and with Broder’s grade II (moderately differentiated). The clinical and histopathological variables did not tend to have an association with infection by the HPV virus.

## Abbreviations

HPV: Human papillomavirus
HU/UFMA: University Hospital of the Federal University of Maranhão
INCA: National Cancer Institute
